# A Novel Finger-Controlled Passive RFID Tag Design for Human–Machine Interaction

**DOI:** 10.3390/s19235125

**Published:** 2019-11-22

**Authors:** Qi Liu, Hui Li, Yu-Feng Yu, Wen-Sheng Zhao, Shuai Zhang

**Affiliations:** 1 School of Automation, Hangzhou Dianzi University, Hangzhou 310018, China; liuqi67@hdu.edu.cn; 2 School of Information and Communication Engineering, Dalian University of Technology, Dalian 116024, China; hui.li@dlut.edu.cn; 3 School of Electronics & Information, Hangzhou Dianzi University, Hangzhou 310018, China; yuyufeng@hdu.edu.cn; 4 Department of Electronic Systems, Aalborg University, 9220 Aalborg, Denmark

**Keywords:** radio frequency identification (RFID), tag antenna, ultra-high frequency (UHF), human–machine interaction (HMI)

## Abstract

Radio frequency identification (RFID) has shown its potential in human–machine interaction thanks to its inherent function of identification and relevant physical information of signals, but complex data processing and undesirable input accuracy restrict its application and promotion in practical use. This paper proposes a novel finger-controlled passive RFID tag design for human–machine interaction. The tag antenna is based on a dipole antenna with a separated T-match structure, which is able to adjust the state of the tag by the press of a finger. The state of the proposed tag can be recognized directly by the code received by the RFID reader, and no complex data processing is needed. Since the code is hardly affected by surroundings, the proposed tag is suitable to be used as a wireless switch or control button in multiple scenarios. Moreover, arrays of the proposed tag with rational tag arrangements could contribute to a series of manual control devices, such as a wireless keyboard, a remote controller, and a wireless gamepad, without batteries. A 3 × 4 array of the finger-controlled tag is presented to constitute a simple passive RFID keyboard as an example of the applications of the proposed tag array and it refers to the arrangement of a keypad and can achieve precise, convenient, quick, and practical commands and text input into machines by pressing the tags with fingers. Simulations and measurements of the proposed tag and tag array have been carried out to validate their performances in human–machine interaction.

## 1. Introduction

Human–machine interaction (HMI) is a process of inputting and outputting information through a human–machine interface, which is a key point of the HMI to achieve the mutual communication, establish the mutual platform between users and machines, and realize the operation on the machines [1].

The most common human–machine interfaces now usually involve traditional devices, such as the keyboard and mouse. In recent years, with the development of recognition technologies, the interaction modes between human and machine have become more diversified, such as vision interaction, voice interaction, and motion interaction [2], and new human–machine interface devices have emerged for these interaction modes. Typical examples include vision interface devices, such as Xbox Kinect [3], PlayStation [4], and PointGrab [5] and motion interface devices, such as wii [6]. However, limitations of high cost, low scalability, limited input accuracy, and need of batteries [7] restrict their large-scale applications. In the next few years, traditional devices constructed to emphasize the transmission of explicit messages [8] will still be the mainstream of human–machine interface devices.

As one of the most important technologies in the Internet of Things, radio frequency identification (RFID) has shown its potential in HMI thanks to its inherent function of identification and extended function of sensing through relevant physical signals [9] during RFID communication. Some RFID tag designs, utilizing the physical signals to obtain HMI input information, have been reported in previous literatures. In [10], a mouth mounted RFID tag, which acted as a tongue touch-controlled switch, was proposed to steer the powered wheelchair by the tongue. Xie et al. [11] proposed a RF (Radio Frequency)-glove, which is composed of five tags, to recognize concurrent multiple finger micromovement. Zou et al. [12] proposed a device-free gesture recognition system by exploiting phase information of RFID signals and Bu et al. [13] turned an ordinary object into a human–machine interface device by attaching a tag array on its surface and tracking its movement by phase variations. In [14], body movement information is collected by the received signal strength (RSS) of RFID tags on different body segments. In [15], real-time activity recognition was achieved by using RFID radio patterns extracted from RSS values of a tag array. In [16], older people falling could be recognized by RSS of RFID tags that were orderly distributed in the room. However, as summarized in Table 1, HMI input information in these RFID tag designs are characterized by physical signals (the measured data) in RFID communication, such as phase and power strength. The physical signals receive serious impact from the environment in measurement, and complex data processing is needed to depict the relationship between the HMI input information and the measured data; therefore, the input accuracy is not desirable due to errors in signal measurement and data processing.

Hsieh et al. proposed touch sensor pads, based on RFID through controlling the on and off state of the tag with finger touch [17], and an RFID contact switch through a magnetic connector coupled with the antenna and the chip [18]. Unlike the HMI tag designs in the literature [10,11,12,13,14,15,16], in these two works, the state of the proposed tag can be recognized directly by the code received by the RFID reader, but not by relevant physical information of signals, while no complex data processing is needed. However, their push/press forces were applied on or very close to the chip, which may easily lead to damaging the chip or a fracture/displacement of the bonding and may greatly affect the durability of tags. Moreover, the contact areas between the finger and the tags in these two works were tiny, which requires an accurate operation on these tags and makes them inconvenient to use.

In this work, we propose a novel finger-controlled ultra-high frequency (UHF) RFID tag design for HMI. The tag antenna consists of a meandered dipole and an impedance matching structure, and a copper patch is placed above the matching structure as a contact switch. The impedance of the tag can be controlled by pressing on the patch. When the patch is pressed, the dipole and the impedance matching structure get connected and the tag can work in the RFID band, then the unique code of its chip will send back to the RFID reader; otherwise, the tag will stop working and the reader could not receive its code. The copper patch has a large contact area and the chip is away from it, which ensures that the patch can still get a good electrical contact with the antenna, even when it has a small displacement with an inclined force, and the chip can keep a long-life durability without the external force.

**Figure 1:**
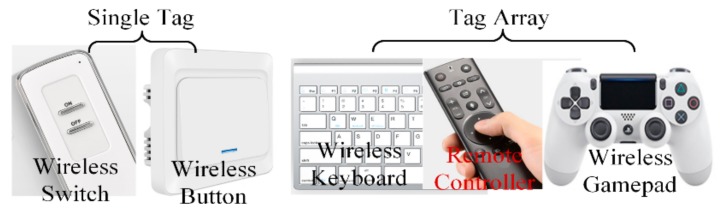
Potential applications of the proposed tag and tag array in smart homes and the industry.

Since the state of the proposed tag can be distinguished by the chip code, which is hardly affected by surroundings, the proposed design can be used as a wireless and passive switch or control button for HMI in multiple scenarios. Moreover, arrays of the proposed tag with rational tag arrangements could contribute to a series of manual control devices for smart homes and industrial applications, such as wireless keyboards, remote controllers, and wireless gamepads, as shown in Figure 1. Compared to the traditional manual control devices, the proposed tag (or tag array) has a longer service time and lower cost, since it needs no batteries or electronic components (except the RFID chip). It is also more portable than the traditional manual control devices thanks to its compact size of 30 mm × 30 mm for every single tag, its light weight of only a few grams, and its flexible structure, which makes it easy to be rolled up after use. More importantly, unlike most traditional wireless manual control devices corresponding to only one transceiver (e.g., a wireless keyboard), a RFID reader could recognize lots of tag arrays, and a tag array can be identified by any authorized reader in an effective range at the same time.

The rest of the paper is organized as follows. Section 2 presents the configuration, mechanism, and optimization of the proposed tag design; a parametric study of the tag design is carried out and the impact of a finger is also taken into consideration. Section 3 proposes a 3 × 4 array of the finger-controlled tag to constitute a simple wireless passive RFID keyboard as an example of the applications of the proposed tag array. The proposed tag array refers to the arrangement of a keypad and can achieve precise, quick, and practical commands and text input into machines by pressing the tags with fingers. In Section 4 we describe the experimental setup and discuss the measurement results of the proposed single tag and tag array. Finally, Section 5 concludes the paper and brings up the improvement design plan for the future.

## 2. Tag Design

### 2.1. Configuration

**Figure 2:**
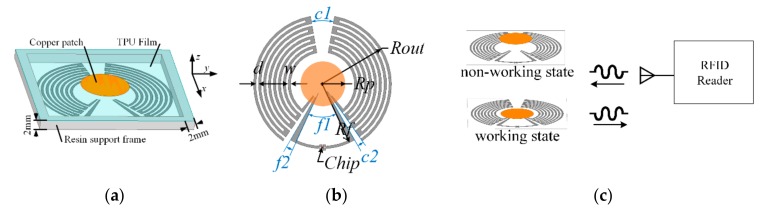
(**a**) Schematic with supporting frame and film; (**b**) geometry without supporting frame and film; (**c**) mechanism of the proposed radio frequency identification (RFID) tag.

The configuration of the proposed tag is depicted in Figure 2. It consists of a disconnected tag and a circular copper patch, as well as the supporting frame and film. The disconnected tag at the bottom is attached to a resin support frame with 2 mm width and 2 mm height, and a flexible thermoplastic polyurethane (TPU) film covers the top surface of the frame. The copper patch is stuck to the internal surface of the film, as shown in Figure 2a.

The disconnected tag is printed on the polyethylene terephthalate (PET, relative permittivity 3.2, loss tangent 0.003) substrate with a thickness of 0.05 mm. It includes two symmetrical arc-shaped meander-line structures and a curved stub between them. These three parts are disjointed because of the existence of two disconnection points. An Impinj Monza 4 RFID chip is placed in the middle of the curved stub, as in Figure 2b.

When the tag is pressed, the patch will be pressed to the tag plane and enabled to connect these two disconnection points. The proposed tag antenna then turns into an arc-shaped meander-line dipole with a T-match configuration [19]. The matching structure, including the curved stub in the middle and the circular copper patch, could be used to adjust the input impedance of the tag antenna. Compared to connecting the antenna with a commercial switch component, the contact structure in this tag features an easier process, a lower cost, and a higher portability at the cost of more stable electrical contact and performance. The geometrical parameters of the proposed tag are presented in Figure 2. A parametric study will be carried out to determine the optimal dimensions in the following.

### 2.2. Mechanism and Analysis

The mechanism of the finger-controlled RFID tag is shown in Figure 2c. In the normal (non-working) state, there was a 2 mm gap between the tag plane and the copper patch. The tag remained disconnected; thus, when the RFID reader sent an interrogating signal, the RFID chip couldn’t be activated and no response signal was received by the RFID reader.

When the patch was pressed to the tag plane and connected the disconnection points of the tag, the tag antenna could work at the UHF frequency band in this working state. When the RFID reader sent an interrogating signal, the RFID chip could be activated by the signal energy received by the tag antenna [20], and a response signal went back to the RFID reader. Since the unique code of the RFID chip was contained in the response signal, we could tell if a proposed tag with a certain RFID chip was pressed by codes received by the RFID reader. Note that when the reader received no response signal with the chip code, it meant that all the tags were in their non-working state at that moment. The proposed tag can be used as a wireless switch or button in this way.

In the working state, the performance of the tag antenna was crucial to the system, as it has a major effect on the reading range of the proposed tag. The impedance of the Impinj Monza 4 RFID chip was (11 − 143j) Ohms at 915 MHz [21]. To achieve a good transmission efficiency, the input impedance of the tag antenna needed to obtain a conjugate matching with the chip. Since external matching networks with lumped components are not feasible due to cost and miniaturization, the matching structure embedded within the tag antenna is an effective solution to the matching problem, as in our proposed tag.

Depending on the structural features of the proposed tag and the equivalent circuit model analysis of the T-match structure in [19], all these structural dimension parameters shown in Figure 2 could be related to one or more of the impedance of the dipole, the impedance of the matching structure, and the current division factor between the dipole and the matching structure, which contribute to the input impedance of the proposed tag antenna in different ways.

### 2.3. Parametric Study

Among all these structural dimension parameters, the *Rout* depended on the size requirement of the tag, and *w*, *d*, *m*, and *f2* were limited by processing precision. Therefore, only parameters *c1*, *c2*, *f1*, *Rf*, and *Rp* were adjustable. These parameters were optimized by the Quasi-Newton optimizer in the Ansoft HFSS software, which was used for all the simulation work in this paper and the parameter studies were carried out in the following ways.

**Figure 3:**
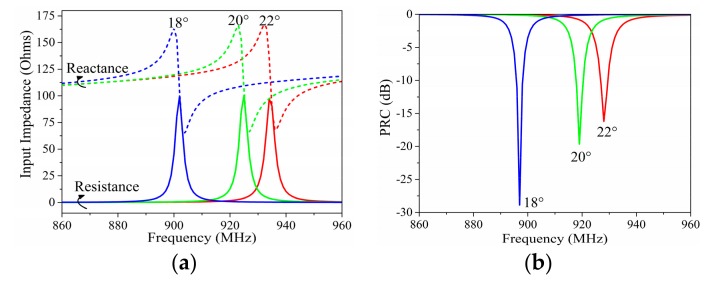
Simulated (**a**) input impedance and (**b**) power reflection coefficient (PRC) results with different angles of *c1*.

Figure 3 presents the variation of input impedance and the power reflection coefficient (PRC) [22] depending on angle *c1*. *c1* directly affected the dipole impedance and consequently changed the input impedance of the tag antenna. The working frequency of the proposed tag moved to the lower frequency with the decrease of *c1*. This movement was nonlinear with the angle decrease, and faster movement appeared in the lower frequency. The decrease of *c1* also slightly increased the input resistance and reactance of the antenna at the same time.

**Figure 4:**
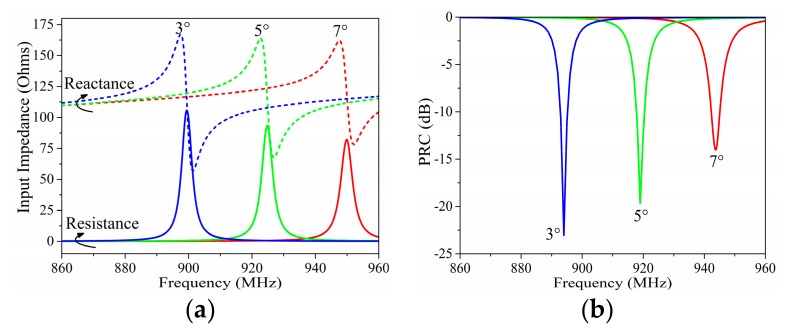
Simulated (**a**) input impedance and (**b**) PRC results with different angles of *c2*.

Figure 4 presents the variation of input impedance and PRC depending on angle *c2*. *c2* affected both the dipole impedance and the current division factor; thus, it showed a different influence on the input impedance from *c1*. The working frequency of the proposed tag moved downward with the decreasing *c2*, but this movement seemed to be linear with the angle decrease. Meanwhile, the decline of *c2* lead to a more significant increase of the input resistance than the input reactance.

**Figure 5:**
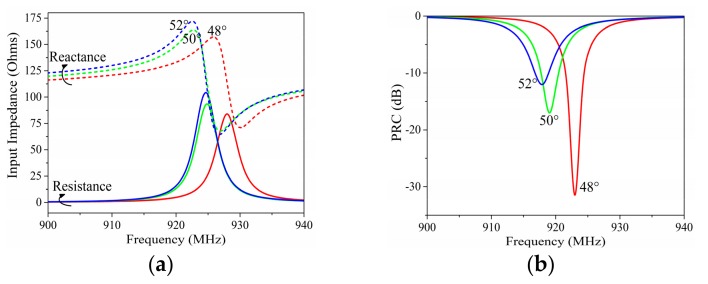
Simulated (**a**) input impedance and (**b**) PRC results with different angles of *f1*.

Figure 5 presents the variation of the input impedance and PRC depending on angle *f1*. *f1* affected both the impedance of the matching stub and the current division factor. It showed a minor influence on the working frequency. However, a larger *f1* lead to the increase of both the input resistance and reactance, and this increase became more significant in the higher frequency.

**Figure 6:**
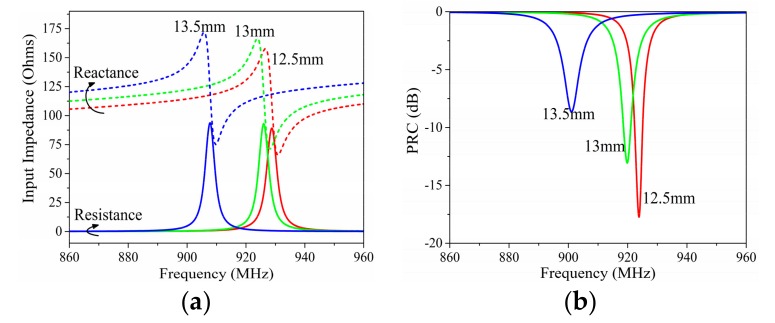
Simulated (**a**) input impedance and (**b**) PRC results with different lengths of *Rf*.

Figure 6 presents the variation of input impedance and PRC depending on length *Rf*. *Rf* affected both the dipole impedance and the current division factor in a different way from angle *c2*. The working frequency of the proposed tag moved to the lower frequency with the increase of *Rf*, and a faster movement appeared in the lower frequency. Meanwhile, the increase of *Rf* lead to a more significant increase of the input reactance.

**Figure 7:**
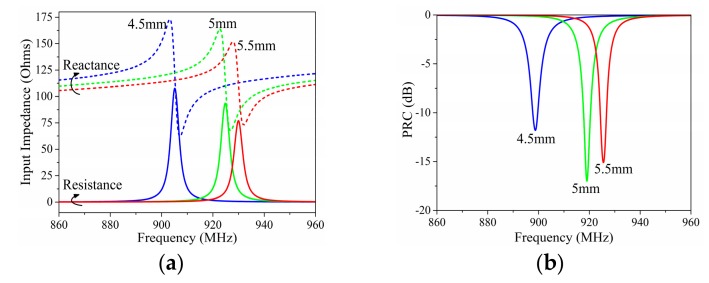
Simulated (**a**) input impedance and (**b**) PRC results with different lengths of *Rp*.

Figure 7 presents the variation of input impedance and PRC depending on length *Rp*. *Rp* affected the dipole impedance, the impedance of the matching stub, and the current division factor at the same time. The working frequency of the proposed tag moved to the lower frequency with the increase of *Rp*, and a faster movement appeared in the lower frequency. At the same time, the increase of *Rp* lead to significant increases of both the input resistance and reactance.

According to size requirements and processing precision, as well as optimization results, the dimensions are determined as in Table 2. The input impedance of the proposed tag was optimized to (9 + 141j) Ω with these dimensions, which was well conjugated when matched to the impedance of the chip (11 − 143j) Ω.

### 2.4. Impact of Finger

**Figure 8:**
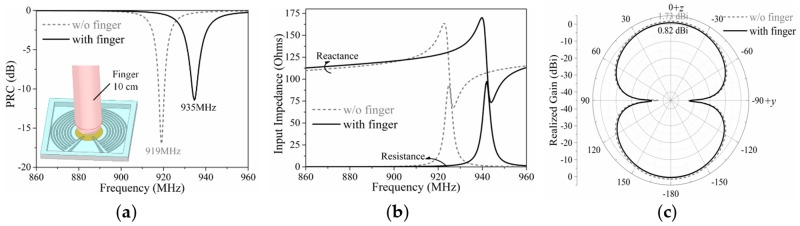
Simulated (**a**) PRC, (**b**) input impedance, and (**c**) radiation pattern results of the optimized proposed RFID tag with/without the impact of the finger.

When the patch was pressed and the tag turned into a working state, the finger on the patch affected the performance of the tag. In order to investigate the impact of the finger, models of the optimized tag in the working state with and without the impact of the finger, as shown in the condensed plot in Figure 8a, were simulated by Ansoft HFSS. The finger was simplified as a cylinder combined with a hemisphere in the tip for faster emulation; the diameters of the cylinder and hemisphere were both 1 cm and the length of the cylinder was 10 cm.

Since the bones make up most of the finger, the relative permittivity of the simplified model of finger was set as 20, which was the approximate relative permittivity of bones at the UHF band. Since the effective conductivity of skin will most likely affect the tag performance, the conductivity of the finger model was set as 1 S/m, which was the approximate value of the skin [23].

The simulation results are presented and compared in Figure 8. The results show that the finger affected the frequency and radiation pattern of the proposed tag at the same time. It shifted the center of working frequency from 919 to 935 MHz and reduced the maximum realized gain from 1.73 to 0.82 dBi. The tag was designed to work in the US standard RFID band by the Federal Communications Commission (FCC band), 902–928 MHz, and with the impact of the finger, the bandwidth of the tag shifted outside this band. Fortunately, the frequency shift was not large enough to stop the tag from working in the FCC band; the proposed tag could still work properly theoretically, even with the impact of the finger.

## 3. Tag Array

Besides using a single finger-controlled tag as a wireless switch or button, the application of the tag array consisting of multiple tags with respective unique codes [24] is more diverse. By rational array arrangement, the tag array can work as a wireless keyboard, remote controller, wireless gamepad, and even more manual devices for HMI. In this section, a simple 3 × 4 tag array was developed as an example of the applications of the proposed tag array. Its performance was simulated and investigated.

### 3.1. Array Structure

**Figure 9:**
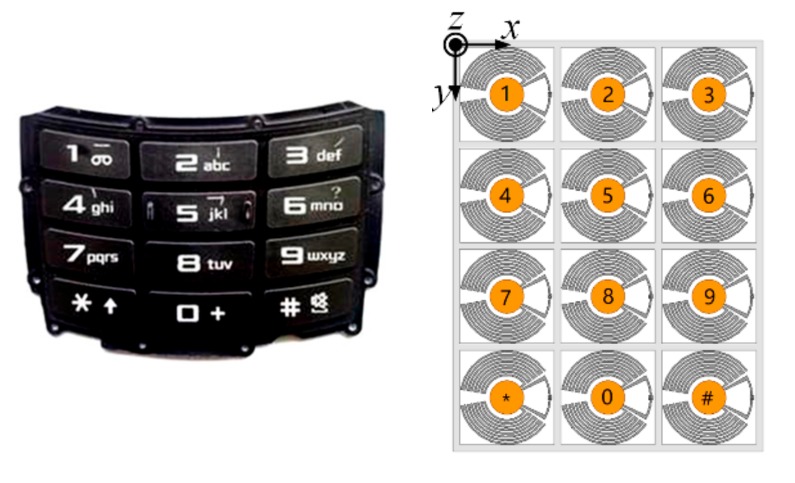
Arrangement of the proposed tag array.

As a trial design, a simple array of the proposed tag is proposed and presented in Figure 9 by referring to a keypad arrangement. It consisted of 3 × 4 tags. Each tag in this array, which may represent a number, a letter, and/or a symbol, could be distinguished by its unique code contained in the response signal back to the reader when it was pressed. Commands and text input to the machine (including computers, phones, and other equipments) could be achieved by switching input methods.

The distance between adjacent tags was 30 mm. The thicknesses and material of the support frame and cover of the tag array were the same as that used in the single tag, and the model of finger was also taken into consideration.

### 3.2. Simulation Results

In the proposed tag array, we supposed that only one tag should be pressed and activated at a time. Since mutual coupling exists between tags in an array, the activated tag at different positions was affected by different adjacent elements. As shown in Figure 9, four typical positions of the activated tag, i.e., position 1, 2, 4, and 5, were considered as they could represent four different neighboring environments of the activated tag in the array. It is worth noting that the finger pressed on the patch may also have an impact on the performance of the activated tag in the array. However, no matter which tag in the array was pressed, the tag performance was required to be robust to guarantee the overall performance of the array. Therefore, the performances of the activated tag at different positions and the impact of finger in this array will be examined in this sub-section.

**Figure 10:**
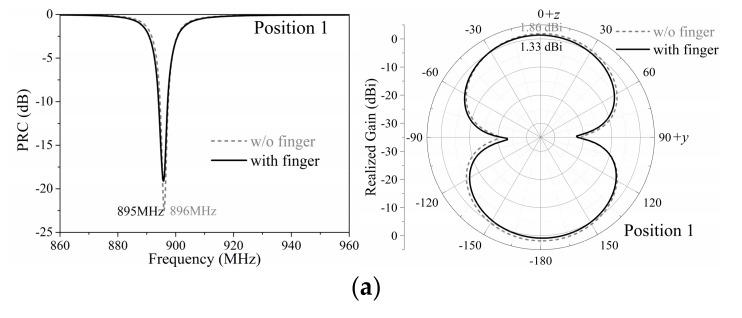

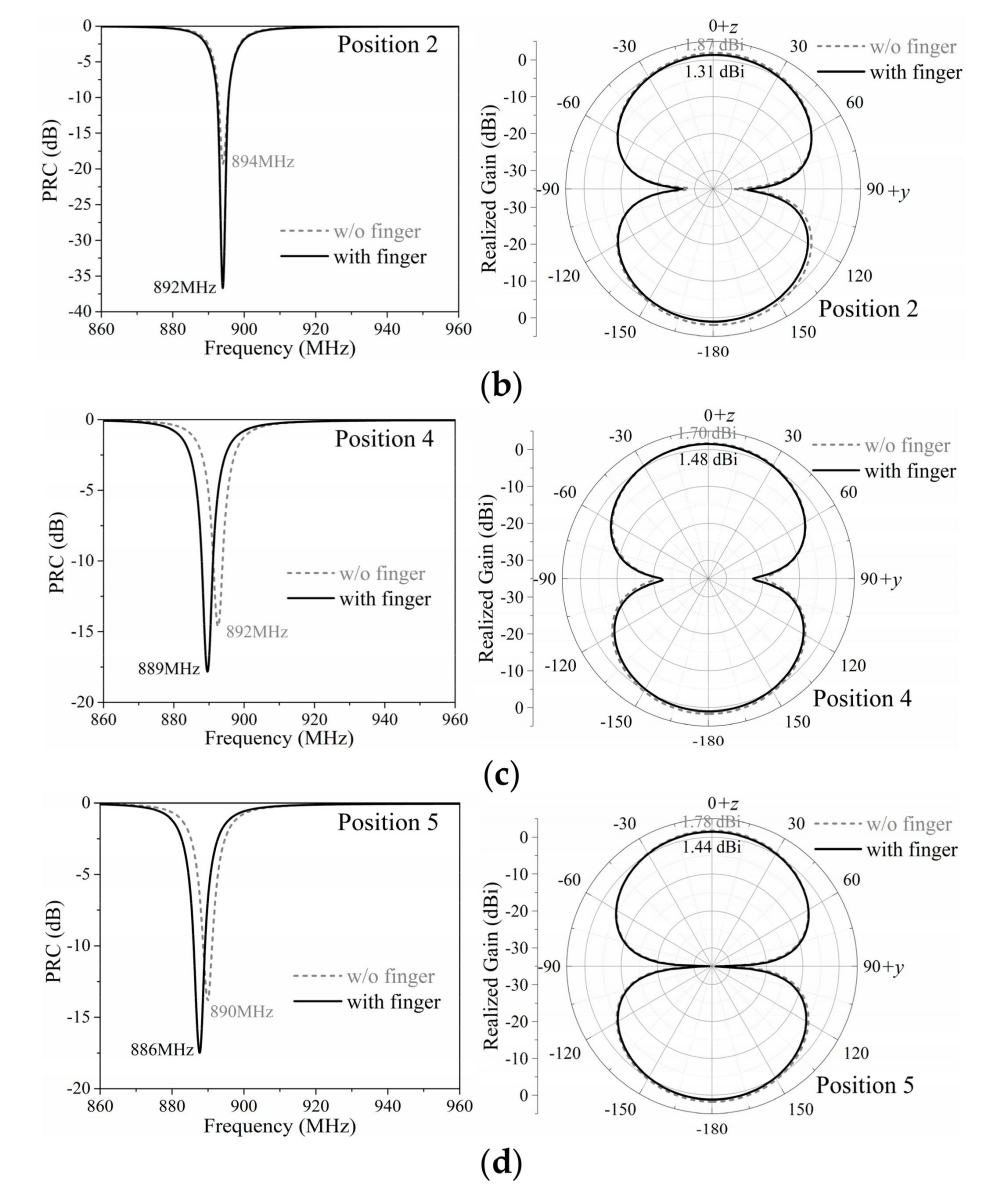
Simulated PRC and radiation patterns in the vertical plane (y–z plane) of the activated tag at (**a**) position 1, (**b**) position 2, (**c**) position 4, and (**d**) position 5 with/without the impact of the finger.

Figure 10 shows the simulation results of PRCs and radiation patterns of activated tags at different positions with and without the impact of the finger. Compared to a single tag, the working frequencies of tags in the array shifted to a lower frequency by about 30 MHz due to existence of adjacent elements. However, the activated tag at different positions had very close performances in both the working frequency and radiation pattern. The finger had a minor influence on the working frequency and realized gain of the tag, which can be attributed to the complex coupling conditions in the array. The center of working frequencies of the activated tag in the array remained between 886–896 MHz regardless of its position and the impact of the finger. The realized gains reduced from 1.8 dBi to about 1.4 dBi at their center frequencies when taking the impact of the finger into consideration; however, this reduction had a slight impact on the performance of the activated tag in the array.

From the simulation results, it can be seen that the performance of tag array was able to remain robust, no matter which tag in this array was pressed and whether the impact of finger was accounted for.

### 3.3. Analysis

Under the hypothesis of polarization matching between the reader and tag antennas, the maximum reading range of RFID tags, *r*, can be calculated by the deformation of the Friis free-space formula, according to the configuration of the reader and the performance of the tag [25]:

(1) $$r=\frac{\lambda }{4\pi }\sqrt{\frac{P_{reader}G_{reader}\tau G_{tag}}{P_{chip}}}$$

where *λ* is the wavelength of the measured frequency, *G_reader_* is the gain of the reader antenna, *P_reader_* is the output power of the reader, *P_reader_G_reader_* is the equivalent isotropic radiated power (EIRP) transmitted by the reader, *τ* is the power transmission coefficient of the tag antenna, *G_tag_* is the gain of the tag antenna, and *P_chip_* is the threshold power of the RFID chip.

According to Equation 1, the reading ranges of tags can be calculated from the simulation results. The theoretical values of reading ranges of a single tag and tags at different positions in the array with 4 W EIRP are listed in Table 3. From the simulation results, we can see that, in theory, the performances of a single tag and tags in an array were very close except for minor frequency deviation.

## 4. Experiments and Discussions

### 4.1. Single Tag Test

**Figure 11:**
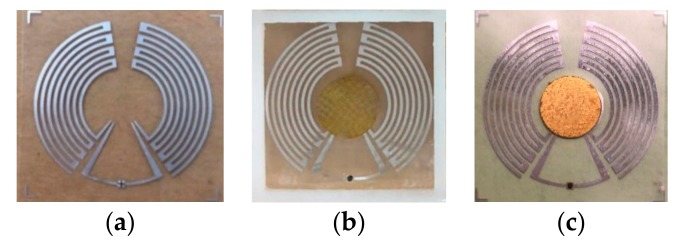
Photographs of the (**a**) disconnected tag, (**b**) finished finger-controlled tag, and (**c**) tag for impedance measurement.

The disconnected tag was fabricated in a roll to roll (R2R) process [26], as shown in Figure 11a. After the attachment of a 3D (three dimensional)-printed resin support frame, the TPU film, and the copper patch, the finished finger-controlled tag is shown in Figure 11b. In addition, since the test cables are sensitive to finger touching, a tag with a copper patch stuck directly on the disconnected tag plane by the silver-epoxy adhesive was used for impedance measurement to avoid additional errors, as in Figure 11c. Since the major structure of the proposed tag adopts the R2R process, the support frame and copper patch of the tag could also be fabricated in mature and cheap processes, such as thermal forming and (computer numerical control (CNC) machining. Therefore, the low cost of the proposed tag can be achieved in large scale processing and production.

The input impedance of the tag antenna was measured by the Rohde & Schwarz ZVA8 vector network analyzer, with the sample in Figure 11c, and the PRC result was derived from the measured impedance. 

**Figure 12:**
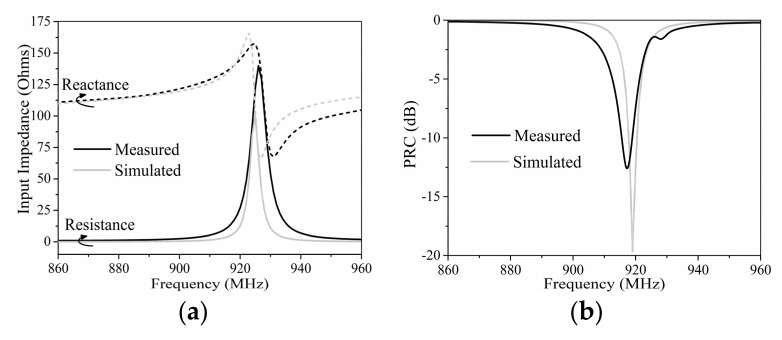
Comparison of the simulated and measured (**a**) input impedance and (**b**) PRC results of the proposed tag antenna without the impact of a finger.

The comparison of simulated and measured impedance and PRC results of the proposed tag are shown in Figure 12. The deviation in the simulated and measured results was supposed to be caused by a manual operation error when applying the silver-epoxy adhesive and stick to the copper patch on the tag plane, and additional resistance was introduced by the silver-epoxy adhesive.

The reading range of the finger-controlled tag was measured by an Impinj R420 [27] RFID reader in the FCC band. The output power from the reader is 1 W (30 dBm), and the reader antenna is Laird S9028PCR, which features a right-hand-circular polarization and a maximum gain of 9 dBi and mainly provides reception and transmission in the FCC band. Since the proposed tag antenna has a linear polarization, a 3 dB loss will be caused by the polarization mismatch between the reader antenna and the tag antenna. Therefore, the equivalent isotropic radiated power (EIRP) is equivalent to 4 W (36 dBm). During the reading range measurement, the tag was attached on a large foam board and the finger pressed vertically on the patch of the tag to avoid additional interference from the hand. The reader antenna and tag antenna kept vertical and their centers keep in the same horizontal line. The measured maximum reading range of the proposed tag with a finger pressing on the copper patch was about 4.5 m.

### 4.2. Tag Array Test

**Figure 13:**
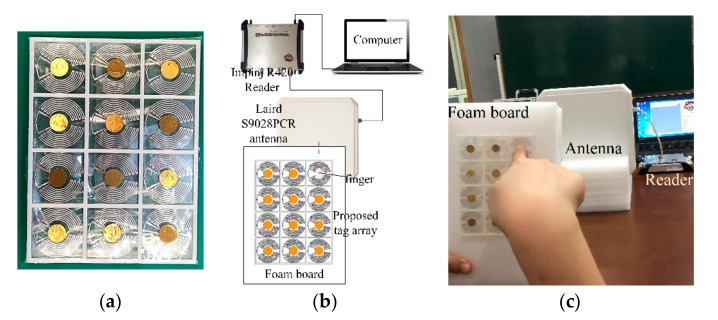
(**a**) Photographs of the finished prototype of the proposed tag array; (**b**) schematic and (**c**) photograph of the measurement of the proposed tag array.

The finished prototype of the proposed tag array is presented in Figure 13a. The measurement setup of the reading ranges of tags in array is shown in Figure 13b. The measurement was taken in a big empty office room of 5 × 6 × 4 m. The measurement equipment was placed on a wooden table in the middle of the room. Similar to the measurement of a single tag, the reading range of the tag in the array was also measured by the Impinj R420 RFID reader. The output power from the reader was 1 W (30 dBm) and the reader antenna was Laird S9028PCR. The reader antenna was fixed vertically by a foam base. The tag array was attached on a large foam board, and the foam board was held by the left hand at its bottom corner to avoid additional interference from the left hand. The tag plane was kept parallel with the reader antenna plane during the measurement, and the tag under test was kept in front of the center of the reader antenna. A finger pressed vertically on the patch of the tag under the test to minimize the impact of the right hand. A picture of the measurement process is shown in Figure 13c. The measured results of tags in the array, as well as the single tags, are represented in Table 4. The reading ranges of tags in the array are 1.3–2.2 m.

From the measured reading ranges in Table 4, we can see that the measured results do not coincide with their theoretical values in Table 3; the measured ranges are much shorter. The main reason for the disparity is the frequency shift. The theoretical values were calculated at their center frequencies, but the measured results were tested in the FCC band. Since the working frequencies of tags in the array shifted to a lower frequency, as in Figure 8 and Figure 10, the power transmission coefficients (*τ* = 1 − PRC) of the tags decrease substantially from their maximum values at the center frequencies to much smaller values in the FCC band. Therefore, the reading ranges of tags in the array measured in the FCC band are much shorter than their theoretical values, according to Equation (1).

Since RFID readers usually do not support a wide-band measurement, the reading range degradation caused by a frequency shift is a common issue in RFID applications, and a targeted optimization for each application scenario is needed to reach its best performance in a specified RFID band. According to the parameter study in the preceding context, the working frequency band of our proposed tag (or tag array) can be conveniently adjusted to fit in any required RFID band by changing the dimensions of the tag, and the reading range can, consequently, be significantly improved.

## 5. Conclusions

This paper proposed a novel passive UHF RFID tag, as well as a tag array, for human–machine interaction. The state of the proposed tag can be recognized directly by the code received by the RFID reader, and no complex data postprocessing is needed. Since the chip code is hardly affected by surroundings, the state recognition of the proposed tag is free from the influence of environment. The single tag can be used as a wireless switch or control button, and the tag array can achieve precise, convenient, quick, and practical commands and text input into machines by pressing the tags with fingers. Additionally, it features a compact size, light weight, flexible structure, long service time, and a low cost.

However, the proposed finger-controlled tag needs to be pressed firmly to contact the copper patch and the tag due to the immature contact design, and the measured reading ranges of the tag and tag array are still not desirable because of the frequency shift issue. Therefore, some developmental research based on this work will be carried out in future, such as an improved tag with a wide band to solve the frequency shift issue and adapt to more scenarios and a finger-controlled tag with better electrical and ergonomic contact design.

## Figures and Tables

**Table 1:** Comparison between the tags in [10,11,12,13,14,15,16] and the proposed tag.

Tag	Input Information	Measured Data	Data Processing
[10]	Tongue Sensing	Backscattered Power	Empirical Induction
[11]	Finger Micromovement	Phase	Filtering and Matching
[12]	Gestures	Phase	Matching Algorithm
[13]	Rigid Transformation	Phase	Filtering and Calculation
[14]	Body Movement	RSS	Machine Learning
[15]	Human Activity	RSS	Recognition Algorithm
[16]	Human Fall	RSS	Machine Learning
Proposed	Working State/Text Input	Chip Code	No

**Table 2:** Dimensions of parameters of the proposed tag antenna.

Parameter	Dimension	Parameter	Dimension
*Rout*	14 mm	*c1*	20°
*w*	0.5 mm	*c2*	5°
*d*	0.5 mm	*f1*	50°
*Rf*	13 mm	*f2*	5°
*Rp*	5 mm		

**Table 3:** Simulation results and calculated reading ranges of single tags and tags in an array (4 W equivalent isotropic radiated power (EIRP); US standard RFID band by the Federal Communications Commission (FCC Band)).

Tag	Center Frequency (MHz)	Max. Realized Gain (dBi)	Calculated Max. Reading Range (m)
Single tag	935	0.82	14.1
Position 1	895	1.33	15.6
Position 2	892	1.31	15.6
Position 4	889	1.48	16.0
Position 5	886	1.44	16.0

**Table 4:** The measured maximum reading ranges of the proposed single tag and tags in the array.

	Position Pressed	Measured Max. Range
Single tag	Tag	4.5 m
Tag array	1	2.2 m
	2	1.9 m
	4	1.4 m
	5	1.3 m
